# Spore Powder of *Ganoderma lucidum* Improves Cancer-Related Fatigue in Breast Cancer Patients Undergoing Endocrine Therapy: A Pilot Clinical Trial

**DOI:** 10.1155/2012/809614

**Published:** 2011-12-10

**Authors:** Hong Zhao, Qingyuan Zhang, Ling Zhao, Xu Huang, Jincai Wang, Xinmei Kang

**Affiliations:** Department of Internal Medicine, The Third Affiliated Hospital of Harbin Medical University, Harbin 150086, Heilongjiang Province, China

## Abstract

The fatigue prevalence in breast cancer survivors is high during the endocrine treatment. However, there are few evidence-based interventions to manage this symptom. The aim of this study was to investigate the effectiveness of spore powder of *Ganoderma lucidum* for cancer-related fatigue in breast cancer patients undergoing endocrine therapy. Spore powder of *Ganoderma lucidum* is a kind of Basidiomycete which is a widely used traditional medicine in China. 48 breast cancer patients with cancer-related fatigue undergoing endocrine therapy were randomized into the experimental or control group. FACT-F, HADS, and EORTC QLQ-C30 questionnaires data were collected at baseline and 4 weeks after treatment. The concentrations of TNF-**α**, IL-6, and liver-kidney functions were measured before and after intervention. The experimental group showed statistically significant improvements in the domains of physical well-being and fatigue subscale after intervention. These patients also reported less anxiety and depression and better quality of life. Immune markers of CRF were significantly lower and no serious adverse effects occurred during the study. This pilot study suggests that spore powder of *Ganoderma lucidum* may have beneficial effects on cancer-related fatigue and quality of life in breast cancer patients undergoing endocrine therapy without any significant adverse effect.

## 1. Introduction

Breast cancer was the most common cancer to affect women [1]. Because of advances in diagnosis and medical treatment of breast cancer, the number of survivors has grown rapidly. These survivors have some conditions and symptoms that can affect not only the quality of their lives, but also their overall survival time [2–4]. One of symptoms which the breast cancer patients often reported is fatigue [5]. Cancer-related fatigue (CRF) is a distressing, persistent, and subjective sense of physical, emotional, and cognitive tiredness related to cancer or cancer treatment that is not proportional to recent activity and interferes with normal functioning [6]. Studies indicate that CRF in breast cancer patients undergoing endocrine therapy could be mediated by the endocrine sequelae of breast cancer treatment, including menstrual disorder, the induction of premature menopause due to ovarian toxicity caused by chemotherapy, radiotherapy, and the side effects of adjuvant endocrine treatment [7–9]. Other potential factors to consider are some physical distress, such as anemia, coronary artery disease, metabolic abnormalities, and emotional distress including anxiety and depression [10, 11].

 The pathophysiologic mechanisms of fatigue in breast cancer patients are not completely understood. Barsevick et al. find that there are many potential mechanisms which can result in CRF. These mechanisms include that cytokine disorder, 5-hydroxy tryptophan (5-HT) neurotransmitter dysregulation, abnormal hypothalamic-pituitaryadrenal (HPA) axis function, and unusual metabolism of adenosine triphosphate (ATP) and muscle. Breast cancer survivors with persistent and gradually aggravating fatigue after treatment had increased levels of immune markers related with proinflammatory cytokine such as interleukin (IL-6) and tumor necrosis factor (TNF-*α*) activity [13–15].

 Despite the prevalence of cancer-related fatigue, there are few evidence-based interventions to manage this symptom. Some researches have examined exercise or psychological treatments for cancer-related fatigue, and these interventions have been shown to reduce fatigue levels and have the evidence support [16–18]. However, survivors with fatigue may be unwilling or unable to go to exercise. Even some breast cancer patients said the more they exercise, the more fatigue they feel. There is also no agreement on the best drug treatment for CRF. The only specific drug recommendation in NCCN is psychostimulant methylphenidate. However, it should be given only under expert supervision and had a potential risk of longer-term addiction [19–21].

 Traditional Chinese medicine can regulate the balance of the body and have fewer adverse reactions if its application is reasonable. Spore powder of *Ganoderma lucidum* (*G. lucidum*) is a kind of Basidiomycete which is a widely used traditional medicine in China [22]. Modern research verified that spore powder of *G. lucidum* has multiple functions, such as blocking histamine release and inhibiting an overstimulated immune system, and has an effect on regulating cellular and humoral immunity [23–25]. Spore powder of *G. lucidum* has many effective components including bioactive compounds like polysaccharides, triterpenoids, alkaloids, enzymes, and proteins [26]. Some researchers find that the triterpenoids from *G. lucidum* suppressed the secretion of inflammatory cytokine TNF-*α* and interleukin-6 and inflammatory mediator nitric oxide (NO) and prostaglandin E2 (PGE2) from lipopolysaccharide-(LPS-) stimulated murine RAW264.7 cells [27].

 On the basis on these research results, spore powder of *G. lucidum* has been suggested as an alternative therapy to control the cancer-related fatigue. The objective of the present study was to evaluate whether spore powder of *G. lucidum *has beneficial effects on fatigue and overall quality of life in patients with breast cancer undergoing endocrine therapy.

## 2. Methods 

### 2.1. Design

The study was performed as a randomized controlled trial comparing an experimental group with control group. Patients in the experimental group were administered with spore powder of *G. lucidum* 1000 mg three times a day for 4 weeks (From Beijing Great Wall Pharmaceutical Factory, Batch number B20050008). The control group received placebo for 4 weeks. At baseline and the end of 4 weeks, functional assessment of cancer therapy-fatigue (FACT-F), hospital anxiety and depression scale (HADS), EORTC quality-of-life questionnaires (QLQ-C30) data were collected; the concentrations of TNF-*α*, IL-6, and liver-kidney function were measured. 

### 2.2. Participants

 The study protocol and all procedures were reviewed and approved by The Institutional Review Board of The Third Affiliated Hospital of Harbin Medical University. All participants were provided written informed consent. These patients were received endocrine therapy for breast cancer at the Third Affiliated Hospital of Harbin Medical University, Harbin, China, between June, 2009 and September, 2010. All patients answered one question each about fatigue and energy. Fatigue was measured with a single item “How much fatigue have you had during the past 4 weeks?” with possible values from 0 (“A great deal of fatigue”) to 10 (“No fatigue at all”). Energy was measured with a single item, “How much energy have you had during the past 4 weeks?” with possible values from 0 (“No energy at all”) to 10 (“A great deal of energy”). The cases were confirmed by a pathological examination; estrogen receptor (ER) and progesterone receptor (PR) statuses were evaluated by standard immunohistochemistry. All patients were ER-positive and PR-positive or PR-negative. The eligibility criteria for study participation were as follows: (a) at least 18 years of age, (b) no documented or observable psychiatric or neurological disorders that would interfere with participation, (c) women diagnosed with stage I-IIIA breast cancer, (d) no history of another cancer, (e) no other chronic or life-threatening diseases in which fatigue is a prominent symptom (e.g., multiple sclerosis, rheumatoid arthritis, fibromyalgia, or chronic fatigue syndrome), (f) previous surgical treatment for breast cancer, (g) completed or undergoing endocrine therapy, (h) endocrine therapy for more than six months, and (i) provision of written informed consent. The exclusion criteria applied to patients who (a) had anemia, defined as hemoglobin level <9 g/dL or patients with platelets <80000/mL, (b) showed levels of abnormal range in serum alanine transaminase (ALT), aspartic acid transaminase (AST), blood urea nitrogen (BUN), or creatinine level, (c) had thyroid disorder with abnormal thyroid stimulating hormone and free T4 level.

### 2.3. Questionnaires Procedures

**FACT-F (Functional Assessment of Cancer Therapy: Fatigue)**

It includes 40 Likert-type items in four scales that assess quality of life in the domains of physical well-being (seven items); social/family well-being (seven items); emotional well-being (six items); functional well-being (seven items); and 13 items in one scale that assesses fatigue. The patients were asked to answer to each item with a score from 0 (not at all) to 4 (very much) [28, 29].

**HADS (The Hospital Anxiety and Depression Scale)**

It consists of two subscales: a seven-item anxiety subscale (HADS-A) and a seven-item depression subscale (HADS-D).The possible scores of both HADS-A and HADS-D range from 0 to 21. A normal value ranges from 0 to 7; a mild disorder ranges from 8 to 10; while scores of 11 or more are considered to be moderate to severe disorder [30]. Some previous studies indicated that the HADS is a valid questionnaire to measure psychological morbidity of Iranian breast cancer patients [31].

**EORTC QLQ-C30 (European Organisation for Research and Treatment of Cancer Core Quality of Life Questionnaire C30)**

It consists of 30 items that reflect the multidimensionality of patients' quality-of-life construct [32]. This questionnaire incorporates five functional scales (physical, role, cognitive, emotional, and social), three symptom scales (fatigue, pain, and nausea and vomiting), 6 single-item measures (dyspnea, insomnia, loss of appetite, constipation, diarrhea, and financial troubles), each item with a score from 1 (not at all) to 4 (very much).It also includes a global health and quality-of-life scale, each item with a score from 1 (not at all) to 7 (very much) [33].

### 2.4. Markers of Fatigue

Blood samples were collected during before and after treatment and were centrifuged to allow serum collection. Serum samples were stored at −80°C. Enzyme-linked immunosorbent assays (ELISA) were used to determine the concentrations of TNF-*α* and IL-6.

### 2.5. Safety and Toxicity

Tests for safety and toxicity included the renal function test (sodium, potassium, and urea creatinine) and liver function test (total protein, albumin, total bilirubin, alkaline phosphate, and alanine transaminase) at baseline and end of the administration period (week 4). The adverse effect was assessed using the National Cancer Institute Common Toxicity Criteria (NCICTC, version 2.0) scale.

### 2.6. Data Analysis

 All analyses were based on data obtained from the aforementioned questionnaires, which were certified by clinical oncologists and by the participants themselves. Demographic variables were analyzed by descriptive statistics to evaluate the clinical and characteristics of the studied samples. *T*-test and linear regression analyses were used. Comparison-of-quality of life scores at different phases of treatment were performed using analysis of variance tests for paired samples. Linear regression models were created using the concentrations of TNF-*α*, IL-6 as the dependent variables, and the variances of cancer-related fatigue as independent variables. Values are expressed as the mean ± standard deviation (mean ± SD) unless stated otherwise, and differences were considered statistically significant at *P* < 0.05. The statistical software SPSS for Windows version 15.0 was used for all statistical analyses. 

## 3. Result

### 3.1. Baseline Characteristics of the Participants

From June 2009 and September 2010, 48 breast cancer patients with cancer-related fatigue undergoing endocrine therapy were randomized into an experimental or a control group. There are 25 patients in an experimental group and 23 in control group. Huang et al. [9] found that BMI, clinical stage, menopausal status, duration of endocrine therapy, physical activity, and diet are associated with fatigue in breast cancer patients undergoing endocrine therapy. The baseline characteristics were not significantly different between the two groups. Baseline demographic and clinical characteristics are described in Table 1.

### 3.2. Outcome Measures

#### 3.2.1. Intervention Effects on Fatigue

Compared with control group, the majority changes of experimental group in FACT-F were observed in the domains of physical well-being (mean, 24.62) and fatigue subscale (mean, 46.78) (*P* < 0.01 and *P* < 0.01, resp.). The emotional (mean, 21.49) and functional well-being (mean, 22.87) of patients improve after intervention (*P* < 0.05 and *P* < 0.05, resp.). The total score of FACT-F was 120.31 (mean) at baseline, and it increased to 141.09 (mean) at the end of 4 weeks in experimental group (*P* < 0.01). No significant difference was between at baseline and at the end of 4 weeks in control group (Table 2).

#### 3.2.2. Intervention Effects on Anxiety and Depression

The significant difference in scores of depression assessment between experimental group and control group was observed at end of 4 weeks (*P* < 0.01). The mean scores of anxiety (4.1) and depression (3.1) assessments in the HADS after treatment were lower than the scores before intervention. Depression of patients in control group at baseline and the end of 4 week had no significant difference (Table 3).

#### 3.2.3. Intervention Effects on Quality of Life

In functioning and global quality-of-life scale in EORTC QLQ-C30, the scores of emotional functioning and cognitive function in the experimental group (the respective mean scores were 79.5 and 75.1) were higher than the outcomes in control group (*P* < 0.05). The domains of physical function (78.2) and global quality of life (68.9) were all significantly improved after 4-week spore powder of *G. lucidum* treatment (*P* < 0.01). No significant change was found in the domains of role functioning and social functioning (Table 4). Compared with control group, the fatigue, sleep disturbance, and appetite loss of patients in experimental group were improved according to the outcomes of the EORTC QLQ-C30 (*P* < 0.01, *P* < 0.01 and *P* < 0.05, resp.) (Table 5).

### 3.3. The Concentration of TNF-*α* and IL-6

**Figure 1:**
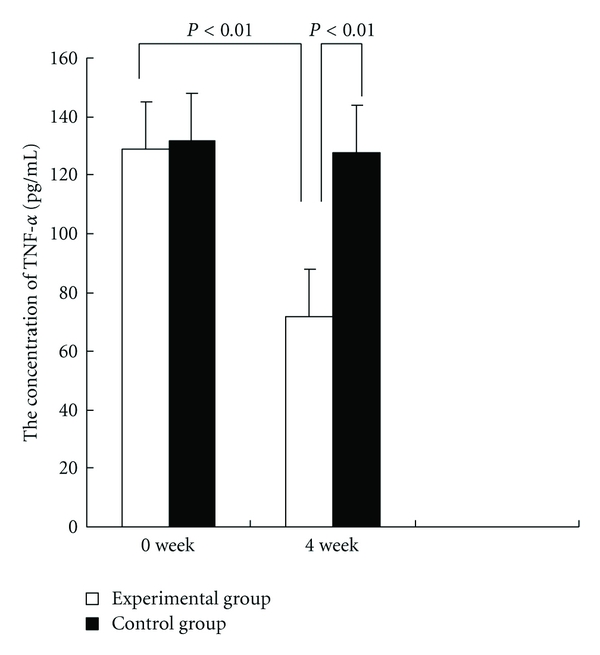
The concentrations of TNF-*α* in experimental group and control group at baseline and 4 week.

**Figure 2:**
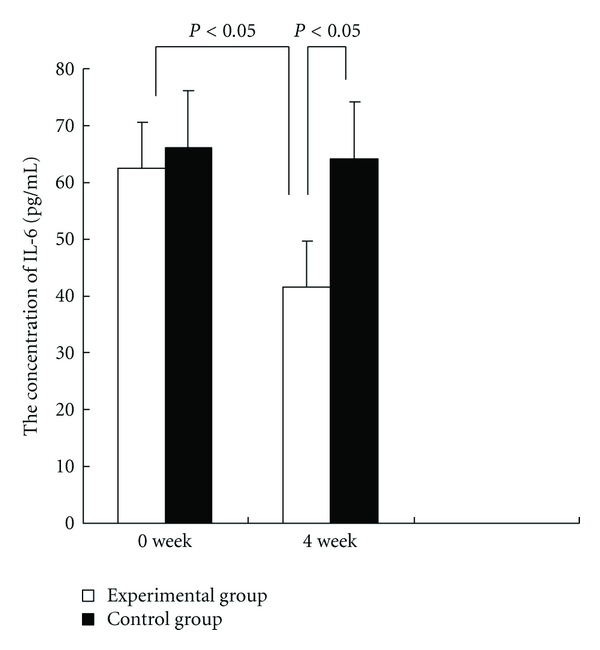
The concentrations of IL-6 in experimental group and control group at baseline and 4 week.

Analysis of the relationship between the concentration of TNF-*α* and the variances of cancer-related fatigue using linear regression found that they have linear correlation. And the concentration of IL-6 has linear correlation with the variances of cancer-related fatigue. In an exploratory analysis of proinflammatory cytokines, markers of cancer-related fatigue, the mean serum concentrations of TNF-*α* pre- and posttreatment in experimental group were 128.70 pg/mL and 71.89 pg/mL. The level of TNF-*α* in serum was significantly lower (*P* < 0.01). The mean serum concentrations of IL-6 pre- and posttreatment in experimental group were 62.43 pg/mL and 37.62 pg/mL. The level of IL-6 in serum was lower (*P* < 0.05) (Figures 1 and 2).

## 4. Toxicity

No serious adverse effects occurred during the study. Only mild discomforts were recorded, and these are shown in Table 6. The two most common discomforts were dizziness (16.0%) and dry mouth (12.0%).

There was no significant change in different parameters of the renal function test (sodium, potassium, and urea creatinine) and liver function test (total protein, albumin, total bilirubin, alkaline phosphate, and alanine transaminase) during the study period. All these parameters were generally within the normal range both before and after study. 

## 5. Discussion

This pilot study suggests that spore powder of *G. lucidum* may have beneficial effects on cancer-related fatigue and quality of life in breast cancer patients undergoing endocrine therapy without any significant adverse effect. The patients who received with spore powder of *G. lucidum* for 4 weeks can decrease degree of fatigue. To our knowledge, this is the first detailed study of adherence to an individualized traditional Chinese medicine intervention targeting generalized cancer-related fatigue during endocrine therapy.

 Endocrine therapy was proposed as a treatment for breast cancer in patients who were with estrogen and/or progesterone receptor-positive tumors. Studies indicate that the fatigue prevalence in patients with breast cancer is high during the endocrine treatment [34]. Fatigue in young breast cancer survivors may experience premature menopause due to estrogen deprivation treatment, thus they experience menopause-related fatigue more earlier in life than those natural menopausal people [35, 36]. Despite exercise or psychological treatments and some medicines used to improve the cancer-related fatigue, these methods may have some shortcomings and deficiencies.

 In this pilot clinical trial, we apply traditional Chinese medicine spore powder of *G. lucidum* to improve cancer-related fatigue. In this study, FACT-F was used to assessing of global fatigue level, and HADS was used to evaluate the anxiety and depression level; quality of life was assessed with EORTC QLQ-C30. From the results of FACT-F, we can find that the scores of physical well-being and fatigue scale change quickly after interventions. These findings are particularly encouraging given that study participants had experienced fatigue for several years before trial onset. However, the score of the social/family well-being was no significant difference between before and after interventions. The results of EORTC QLQ-C30 in this study showed significant improvements in physical functioning, emotional functioning, and cognitive functioning of patients. These findings suggest that the intervention may have beneficial effect on strength in addition to subjective symptoms. Meanwhile, the symptoms of sleep disturbance and appetite loss decreased with improving cancer-related fatigue. Anxiety and depression scores declined significantly over the study, indicating a possible improvement in anxiety and depression. One explanation for the improvement in depression is that with less fatigue and more energy, these breast cancer survivors were able to do some activities that they previously had given up. These activities brought pleasure into their lives.

 The pharmacological effect of spore powder of *G. lucidum* is based on their powerful immune-modulating action and immune potential capability. The active elements of it downregulated the expression of iNOS and COX-2, suppressed the release of TNF-*α* and IL-6, and suppressed the LPS-dependent activation of NF-*κ*B in RAW264.7 cells [27, 37]. From the ELISA consequence of cytokines as markers of cancer-related fatigue, the concentrations of TNF-*α* and IL-6 after treatment were significant lower than these before treatment. 

 In this 4-week study, spore powder of *G. lucidum* was well tolerated. No serious adverse effects occurred during the study. Most side effects were mild dizziness. At the end of 4 weeks, the levels of ALT, AST, BUN, and creatinine were no significant difference than these at baseline in statistics.

 Although these results are promising, the study has limitations that render the findings preliminary. The first is the small sample size; only 48 women participated in the intervention. Therefore, a sampling bias does exist. In addition, it is possible that nonspecific aspects of the intervention (e.g., social support) may have influenced fatigue scores.

 Despite these limitations, our data suggest that spore powder of *G. lucidum* is a well-tolerated option for the treatment of cancer-related fatigue. In addition, the improvements in emotional functioning seen in our study suggest that it has a place for the treatment of depression in patients whose treatment with traditional antidepressants impractical. More rigorous trials are needed to confirm the efficacy of Spore of *G. lucidum*.

## 6. Conclusion

Cancer-related fatigue and poor quality of life are serious problems that significantly affect the morbidity of breast cancer patients. Spore of *G. lucidum* may offer a useful complementary treatment that can improve the situations in the experimental group. This pilot clinical trial is the first step to explore the efficacy of herbal therapy on fatigue and quality of life in breast cancer patients. Results from the studies could provide evidence for efficacy and might be used to design more future comprehensive studies.

##  Conflict of Interests

The authors declare that they have no conflict of interests.

## Figures and Tables

**Table 1:** Demographics of patients in each group.

	Experimental group (*n* = 25)	Control group (*n* = 23)	*P*-value
(1) age (year)			
mean (SD)	51.3 (9.8)	53.2 (8.7)	*P* > 0.1

(2) Body mass index (kg/m^2^)			
mean (SD)	22.9 (3.8)	23.2 (3.5)	*P* > 0.1

(3) Menopausal status *n* (%)			*P* > 0.05
Pre-menopause	10 (40.0%)	12 (52.1%)	
Post-menopause	15 (60.0%)	11 (47.9%)	

(4) Clinical stage *n* (%)			*P* > 0.05
Stage I	8 (32.0%)	7 (30.4%)	
Stage II	10 (40.0%)	9 (39.2%)	
Stage IIIA	7 (28.0%)	7 (30.4%)	

(5) Treatment *n* (%)			*P* > 0.05
Surgery + endocrine therapy	4 (16.0%)	3 (13.1%)	
Surgery + radiation + endocrine therapy	4 (16.0%)	4 (17.3%)	
Surgery + chemotherapy + endocrine			
Therapy	11 (44.0%)	10 (43.5%)	
Surgery + chemotherapy + radiation + endocrine therapy	6 (24.0%)	6 (26.1%)	

(6) Duration of endocrine therapy *n* (%)			*P* > 0.05
>6 Months to 3 years	15 (60.0%)	13 (56.5%)	
>3 years to 5 years	10 (40.0%)	10 (43.5%)	

(7) Physical Activity *n* (%)			*P* > 0.05
MHW < 3.3	2 (8.0%)	1 (4.3%)	
3.3 ≤ MHW < 10.0	7 (28.0%)	10 (43.5%)	
10.0 ≤ MHW < 20.0	12 (48.0%)	9 (39.1%)	
MHW ≥ 20.0	4 (16.0%)	3 (13.1%)	

(8) Diet *n* (%)			*P* > 0.05
0	3 (12.0%)	2 (8.6%)	
1~2	7 (28.0%)	6 (26.1%)	
3~4	8 (32.0%)	9 (39. 2%)	
5	7 (28.0%)	6 (26.1%)	

**Table 2:** FACT-F scores for experimental group and control group.

Subscal (range of scores)	Experimental group (*n* = 25)	Control group (*n* = 23)
Physical (0–28)		
Week 0	20.35 ± 4.07	19.43 ± 4.19
Week 4	24.62 ± 3.27^∗∗##^	20.65 ± 3.97

Social/family (0–28)		
Week 0	21.35 ± 3.91	20.89 ± 3.91
Week 4	22.37 ± 3.61	21.12 ± 4.07

Emotional (0–24)		
Week 0	17.61 ± 4.00	16.73 ± 3.87
Week 4	21.49 ± 2.21^∗#^	17.99 ± 2.07

Functional (0–28)		
Week 0	17.87 ± 4.93	17.35 ± 4.87
Week 4	22.87 ± 5.13^∗#^	18.29 ± 3.79

Fatigue subscale (0–52)		
Week 0	39.76 ± 5.10	40.35 ± 6.10
Week 4	46.78 ± 5.07^∗∗##^	40.92 ± 5.62

Total (0–160)		
Week 0	120.31 ± 20.15	119.65 ± 18.99
Week 4	141.09 ± 17.23^∗∗# #^	121.01 ± 19.13

*P < 0.05 versus week 0 of experimental group.

**P < 0.01 versus week 0 of experimental group.

^#^ *P* < 0.05 versus week 4 of control group.

^##^ *P* < 0.01 versus week 4 of control group.

**Table 3:** HAD scores for experimental group and control group.

Subscale	Experimental group (*n* = 25)	Control group (*n* = 23)
Anxiety		
Week 0	6.3 ± 3.2	6.5 ± 3.4
Week 4	4.1 ± 2.9^∗#^	6.1 ± 3.2

Depression		
Week 0	4.9 ± 3.8	4.8 ± 3.1
Week 4	3.1 ± 2.8^∗∗##^	4.6 ± 2.9

Total		
Week 0	10.9 ± 4.1	10.8 ± 3.9
Week 4	7.1 ± 3.1^∗∗##^	9.8 ± 3.4

*P < 0.05 versus week 0 of experimental group.

**P < 0.01 versus week 0 of experimental group.

^#^ *P* < 0.05 versus week 4 of control group.

^##^ *P* < 0.01 versus week 4 of control group.

**Table 4:** Patients' functioning and global quality of life scores as measured by the EORTC QLQ-C30.

Subscale	Experimental group (*n* = 25)	Control group (*n* = 23)
Functional scales		
Physical functioning		
Week 0	63.7 ± 25.9	64.0 ± 27.1
Week 4	78.2 ± 26.1^∗∗##^	64.5 ± 28.7

Role functioning		
Week 0	65.2 ± 33.6	68.1 ± 31.2
Week 4	79.1 ± 31.1	69.3 ± 32.4

Emotional functioning		
Week 0	61.3 ± 30.2	62.2 ± 32.5
Week 4	79.5 ± 31.5^∗∗##^	64.3 ± 31.8

Social Functioning		
Week 0	72.4 ± 29.1	74.3 ± 28.6
Week 4	76.1 ± 26.8	75.5 ± 27.9

Cognitive functioning		
Week 0	65.6 ± 24.3	66.4 ± 23.2
Week 4	75.1 ± 26.5^∗#^	68.3 ± 26.3

Global Quality of Life		
Week 0	55.8 ± 22.9	56.6 ± 23.0
Week 4	68.9 ± 21.4^∗∗##^	57.7 ± 24.2

*P < 0.05 versus Week 0 of experimental group.

**P < 0.01 versus Week 0 of experimental group.

^#^ *P* < 0.05 versus week 4 of control group.

^##^ *P* < 0.01 versus week 4 of control group.

**Table 5:** Patients' symptom scores on the EORTC QLQ-C30.

Symptom scales	Experimental group (*n* = 25)	Control group (*n* = 23)
Fatigue		
Week 0	43.7 ± 17.9	42.3 ± 15.7
Week 4	31.1 ± 18.1^∗∗##^	40.2 ± 16.8

Pain		
Week 0	32.4 ± 12.7	31.3 ± 13.6
Week 4	29.3 ± 14.6	30.7 ± 17.3

Sleep disturbance		
Week 0	56.5 ± 21.8	55.8 ± 22.6
Week 4	42.3 ± 26.2^∗∗##^	53.9 ± 24.8

Appetite loss		
Week 0	32.5 ± 19.3	32.3 ± 17.4
Week 4	24.3 ± 18.4^∗#^	30.3 ± 16.5

Constipation		
Week 0	31.1 ± 11.4	32.5 ± 12.8
Week 4	28.2 ± 13.3	30.6 ± 14.7

Diarrhea		
Week 0	12.9 ± 10.9	12.7 ± 10.5
Week 4	11.8 ± 8.8	10.6 ± 9.6

*P < 0.05 versus week 0 of experimental group.

**P < 0.01 versus week 0 of experimental group.

^#^ *P* < 0.05 versus week 4 of control group.

^##^ *P* < 0.01 versus week 4 of control group.

**Table 6:** Mild adverse events of patients in Experimental Group.

Adverse events	Cases (%)
Dizziness	4 (16.0%)
Dry mouth	3 (12.0%)
Diarrhea	2 (8.6%)
Stomach discomfort	2 (8.6%)
Nausea	2 (8.6%)
Epistaxis	1 (4.0%)
Sore throat	1 (4.0%)
